# The burden of disease in Saudi Arabia 1990–2017: results from the Global Burden of Disease Study 2017

**DOI:** 10.1016/S2542-5196(20)30075-9

**Published:** 2020-05-19

**Authors:** GBD 2017 Saudi Arabia Collaborators, Stefanos Tyrovolas, Charbel El Bcheraoui, Suliman A Alghnam, Khalid F Alhabib, Majid Abdulrahman Hamad Almadi, Rajaa M Al-Raddadi, Neeraj Bedi, Maha El Tantawi, Varsha Sarah Krish, Ziad A Memish, Yousef Mohammad, Alex Molassiotis, Demosthenes Panagiotakos, Nasir Salam, Badr Hasan Sobaih, Ali H Mokdad

## Abstract

**Background:**

Availability of data to assess the population health and provision and quality of health care in Saudi Arabia has been lacking. In 2010, Saudi Arabia began a major investment and transformation programme in the health-care sector. Here we assess the impact of this investment era on mortality, health loss, risk factors, and health-care services in the country.

**Methods:**

We used results of the Global Burden of Diseases, Injuries, and Risk Factors Study (GBD) 2017 to describe the levels and temporal patterns in deaths, health loss, risk factors, and health-care access and quality in the Saudi Arabian population during 1990–2010 (before the health-care investments and reform) and 2010–17 (during health-care investments and reform). We also compared patterns in health outcomes between these periods with those in the north Africa and the Middle East GBD region and the Gulf Cooperation Council countries.

**Findings:**

Age-standardised mortality in Saudi Arabia decreased from 1990 to 2010 (annualised rate of change of −0·58%), and this decrease was further accelerated from 2010 to 2017 (–2·20%). The north Africa and the Middle East GBD region also had decreases in mortality during these periods, but for 2010–17 the decrease was not as low as in Saudi Arabia (–1·29%). Transport injuries decreased from third ranked cause of disability-adjusted life-years in 2010 to fifth ranked cause in 2017 in Saudi Arabia, below cardiovascular diseases (ranked first) and musculoskeletal disorders (ranked second). Years lived with disability (YLDs) due to mental disorders, substance use disorders, neoplasms, and neurological disorders consistently increased over the periods 1990–2010 and 2010–17. Between 1990 and 2017, attributable YLDs due to metabolic, behavioural, and environmental or occupational risk factors remained almost unchanged in Saudi Arabia, with high body-mass index, high fasting plasma glucose concentration, and drug use increasing across all age groups. Health-care Access and Quality (HAQ) Index levels increased in Saudi Arabia during this period with similar patterns to the rest of the Gulf Cooperation Council countries and the north Africa and the Middle East GBD region.

**Interpretation:**

Decreases in mortality continued at greater rates in Saudi Arabia during the period of 2010–17 than in 1990–2010. HAQ Index levels have also improved. Public health policy makers in Saudi Arabia need to increase efforts to address preventable risk factors that are major contributors to the burden of ill health and disability.

**Funding:**

Bill & Melinda Gates Foundation.

## Introduction

Demographic data suggest that the world's population is changing, metabolic morbidities have spread from western populations to populations around the world and older populations (aged ≥60 years) are comprising a considerable proportion of the population. Countries in north Africa and the Middle East are having a similar transition, with substantial social and health implications.^1^, ^2^, ^3^ Saudi Arabia has the fastest growing population among the Gulf Cooperation Council countries, projected to reach more than 35 million people by 2025.^4^ Almost 60% of the Saudi Arabian population is aged 35 years or younger and the demand for health-care services is steadily increasing.^4^

Starting in 2010, Saudi Arabia increased investments in health care, with an increase in domestic general government health expenditure (percentage of Gross Domestic Product [GDP]) between 2011 and 2015 of almost 68%, much higher than the north Africa and the Middle East GBD region's increase (19·2%) and the highest in 2015 among the Gulf Cooperation Council countries.^5^ In 2016, as a part of Vision 2030, the ministry of health announced the transformation of the health-care system, including three major objectives (ease access to health services, improve quality and efficiency of health-care services, and promote prevention against health risks) focusing on improving preventive and therapeutic health-care services. In support of this action, in 2012 the Saudi Arabian ministry of health started a collaboration with the Institute for Health Metrics and Evaluation (IHME), which coordinates the GBD study, to better explore and understand the health of Saudi Arabia's population through the Saudi Health Interview Survey.^6^, ^7^

Many countries in the greater north Africa and the Middle East region share important health-determining factors, including religion and similar ethnicity. However, substantial disparities in health indicators have been observed among these countries. Focusing specifically on Saudi Arabia, results from the Saudi Health Interview Survey have reported a high burden of nutrition-related and lifestyle-related risk factors such as obesity, hypertension, and diabetes.^6^ Substantial increases in the burden attributable to risk factors have been reported specifically among young Saudi Arabians (aged 15–24 years).^8^ In 2010, a GBD country-specific report for Saudi Arabia reported that the country also faces an increased epidemic of non-communicable diseases and road and transportation injuries.^9^ The main causes of death were reported to be ischaemic heart disease, stroke, and road injuries. Depression was the leading cause of disability in females, and road injuries were the leading cause in males. High body-mass index (BMI) has been reported to be a major risk factor for disease burden among the Saudi population.^9^

**Research in context**

**Evidence before this study**

In 2010, Saudi Arabia began a health-care investment and transformation era and plans to continue in this direction with a transformation programme announced by the ministry of health in 2016, Vision 2030. A previous Global Burden of Disease (GBD) country-specific report for Saudi Arabia reported the population's disease burden up to 2010. To better understand the current state of public health in Saudi Arabia and its trajectory before and after 2010 requires comprehensive evaluation of multiple health-related domains, including all-cause and cause-specific mortality, non-fatal health loss, risk factors, and health-care services.

**Added value of this study**

This study is the first to report the effects of the health-care investment programme on the health of Saudi Arabia's population using data from the Global Burden of Diseases, Injuries, and Risk Factors (GBD) study 2017. We did a comprehensive analysis of annual changes in population health in Saudi Arabia from 1990 to 2017 using comparators in the geographical region that allowed us to capture region-specific divergent temporal trends, and we assessed these trends in conjunction with the Healthcare Access and Quality Index. The analysis of these data sources enabled us to identify evidence of changes in the health of the Saudi population compared with regional populations (the north Africa and Middle East GBD region and Gulf Cooperation Council countries) from 1990 to 2010 with those from 2010 to 2017.

**Implications of all the available evidence**

We found mortality decreased in Saudi Arabia for 1990–2010, which was accelerated in 2010–17, with similar trajectories to the countries of the north Africa and Middle East GBD region and the Gulf Cooperation Council. Although health-care access and quality have improved in Saudi Arabia, the disease burden due to mental disorders and substance use disorders has increased, as has the population's morbidity due to lifestyle and metabolic risk factors. Our findings suggest the need for further enhancement of public health and preventive policies in Saudi Arabia.

Until now, availability of data to assess the Saudi Arabian population's state of health and the provision and quality of health care since the ministry of health's investments started in 2010 has been lacking.^10^, ^11^ The Saudi Health Interview Survey is one of the recent efforts undertaken with nationally representative data that has reported preliminary results.^6^ Documenting the impact of diseases and conditions on population health is demanding from the standpoint of both surveillance and analytical methods. The only comprehensive effort to quantify global population health is the Global Burden of Disease study, which offers a suitable methodology to compare between different locations and time periods. In this Article, given the scarcity of analysis of the disease burden and data on the quality of and access to health care in Saudi Arabia, we used GBD data to explore levels and temporal trends in mortality, health loss, risk factors, and health-care services in Saudi Arabia from 1990 to 2017, comparing levels and trends during the periods before (1990–2010) and after (2010–17) health investments. Additionally, we compared Saudi Arabia's trends in health outcomes during these two periods with those of the north Africa and the Middle East GBD region and the Gulf Cooperation Council countries.

## Methods

### Locations and metrics

GBD 2017 quantified multiple measures of health loss for 282 causes of death, 359 diseases, and 84 risk factors for each of 195 countries and territories, 23 age groups, and both sexes, from 1990 to 2017.^12^ The GBD study is organised with geographical categorisation of super-regions and regions, with 195 countries and territories assigned within these areas. Within this categorisation, the GBD study estimates fatal and non-fatal causes in a hierarchy of levels, with three at Level 1, 22 at Level 2, 169 at Level 3, and 293 at Level 4. From Level 1 to Level 4, the causes and risk factors are clustered from less to more detail. For the present analysis, we compared Saudi Arabia with the GBD region of north Africa and the Middle East (including Afghanistan, Algeria, Bahrain, Egypt, Iran, Iraq, Jordan, Kuwait, Lebanon, Libya, Morocco, Oman, Palestine, Qatar, Saudi Arabia, Sudan, Syria, Tunisia, Turkey, the United Arab Emirates, and Yemen), and with the countries of the Gulf Cooperation Council (ie, Bahrain, Kuwait, Oman, Qatar, Saudi Arabia, and the United Arab Emirates). GBD results are available publicly for visualisation and for download online. By comparing Saudi Arabia with the north Africa and the Middle East region and Gulf Cooperation Council countries, we aim to show the differences in the disease burden in Saudi Arabia compared with other countries in these regions, such as has been done with Greece in previous GBD publications.^13^

In this study we used several metrics depending on the specific issue being assessed. We compared mortality across locations to control for population size, we used age standardisation to control for age structure, and comparative rankings and disease rates for age groups 15–49 years and 50–69 years to examine specific causes and risk factors for different age groups. We compared trends using the annualised rate of change, calculated as the final estimates, divided by initial estimates, then divided by number of years, using the natural log transformation of this figure. Annualised rates of change have been estimated for the periods 1990–2010 and 2010–17. Detailed methods for the overall study and for each specific cause and risk are available in GBD 2017 summary publications,^12^, ^14^ each of which is compliant with the Guidelines for Accurate and Transparent Health Estimates Reporting (GATHER). Here we briefly describe our methods for deriving each of the GBD metrics included in the present analysis, including deaths, health expenditure, years of life lost (YLLs), years lived with disability (YLDs), disability-adjusted life-years (DALYs), and the Healthcare Access and Quality (HAQ) Index. However, notably, GBD 2017 data include actual local population information data and are missing expatriates' health-related information.

The GBD protocol has been approved by the research ethics board at the University of Washington.

### All-cause mortality and causes of death

Mortality estimation methods are described in detail elsewhere.^12^ Briefly, we estimated all-cause mortality primarily from vital registration data, adjusted for completeness in Saudi Arabia. We used spatiotemporal effects and covariates to extend estimates to 2017. Under-5 mortality and mortality for populations aged 15–59 years were estimated separately and linked using empirical life tables to derive age-sex-specific all-cause mortality. The lowest observed risk of death for each age group in total populations greater than 5 million people were summed to construct a global standard life expectancy.

We estimated cause-specific deaths and YLLs for underlying causes of mortality, ensuring that the sum of all specific causes was equal to all-cause mortality. We assigned each death to a single cause. We adjusted vital registration and census death data for incompleteness and misclassification (eg, prostate cancer in a female); non-specific and intermediate codes (eg, sepsis, heart failure, unknown causes) were redistributed using age-specific, sex-specific, and geography-specific statistical redistribution methods before modelling. The estimation methods we used most commonly was Cause of Death Ensemble modelling (CODEm). CODEm uses a train-test-test approach, first testing all combinations of selected country-level covariates and their relationship to in-sample data, then ranking those component models on the basis of out-of-sample predictive validity to construct weighted ensembles. All ensemble models are then ranked again on a second round of out-of-sample predictive validity to select a final model. We multiplied cause-specific fractions by all-cause mortality estimates to calculate cause-specific deaths, the results of which were then scaled with all other causes to match all-cause mortality. In this study, we assigned Level 2 causes of death in our analysis. We calculated YLLs by multiplying age-specific deaths by global standard life expectancy at age of death.

### Non-fatal health loss as expressed by YLDs and DALYs

We used epidemiological data from systematic literature reviews, health surveys, surveillance systems, disease registries, and hospital and claims databases to generate cause-specific and sequela-specific prevalence and incidence estimates. We generated these estimates using a variety of modelling approaches, of which Bayesian meta-regression compartmental modelling in DisMod-MR 2.1 was the most common.^15^ We derived disability weights for each unique health state from population surveys of over 60 000 respondents completed for GBD 2010 and GBD 2013.^16^, ^17^ We then used a microsimulation framework to adjust for comorbidities and calculated YLDs for each cause by multiplying prevalence and corresponding disability weights for each sequela of each cause.^18^ In this study for Saudi Arabia, we assigned Levels 2, 3, and 4 causes of YLDs in our analysis.

DALYs are a summary measure of health that we calculated for each age-sex-year-state-cause strata by summing the fatal (YLL) and non-fatal (YLD) components.^18^ In this study for Saudi Arabia, we assigned Level 2 causes of DALYs in our analysis.

### Risk factor estimation

The GBD 2017 comparative risk assessment framework classified each of the 84 risk factors and clusters of risk factors into one of three categories: behavioural, environmental or occupational, or metabolic.^19^ We identified, evaluated, and modelled data on exposure levels for each risk factor using similar approaches to non-fatal models, with added emphasis on accurately fitting distributions of exposure for continuous and polytomous risk factors. We estimated quantitative relative risk (RR) for each risk-outcome pair and calculated population attributable fraction statistics using standard GBD comparative risk assessment methods.^14^ In this study, we applied Level 1 and 2 risks for the attributable risk factor analysis.

Due to the high proportion of younger people (ie, aged 15–49 years) in the Saudi Arabian population, we also stratified the analysis of risk factors and disability burden by the age groups of 15–49 and 50–69 years, in three time periods (1990, 2010, and 2017). For these age groups, we applied a Level 4 analysis of the attributable risk factors.

### Healthcare Access and Quality (HAQ) Index

The HAQ Index is a composite metric that was developed after GBD 2015.^20^ It is based on comparative mortality rates for health-care-sensitive diseases, standardised to risk exposure level, and is meant to quantify the overall performance of health systems.^21^ HAQ Index was calculated using principal components analysis and scales from 0 to 100. Data were available from 1990 to 2016. Here we analyse and compare the trajectories of HAQ Index for Saudi Arabia, countries in the Gulf Cooperation Council, and the north Africa and Middle East GBD region between 1990 and 2016.

### Uncertainty analysis

We derived uncertainty for each metric from 1000 draws from the distribution of each estimation step by age, sex, and location for each year included in the GBD 2017 analysis; lower and upper uncertainty intervals (UIs) are the ordinal 25th and 975th draws of each quantity—ie, 95% UIs. UIs allow final estimates to reflect the combined uncertainty of multiple modelling steps. 95% UIs for mortality and YLLs show uncertainty in regression coefficients, due to sampling and non-sampling error in the cause of death data, due to various model specifications, and in the Levels of all-cause mortality. 95% UIs for YLDs show uncertainty in prevalence estimates, distribution of severity within each cause, and disability weight valuations. We aggregated uncertainty across age, sex, and location on each draw assuming no correlation.^22^

### Role of the funding source

The funder of the study had no role in study design, data collection, data analysis, data interpretation, or writing of the report. The corresponding author had full access to all the data in the study and had final responsibility for the decision to submit for publication.

## Results

Age-standardised all-cause mortality in Saudi Arabia was 634·90 (95% UI 595·69 to 694·39) deaths per 100 000 population in 2017 compared with 740·80 (684·43 to 789·93) deaths per 100 000 population in 2010 and 832·83 (743·47 to 936·51) per 100 000 population in 1990, corresponding to a −0·58% annualised rate of change from 1990 to 2010, and a −2·20% annualised rate of change for 2010 to 2017; an almost five times higher annualised rate of change during the period after health-care investment and reformation (table). The decrease in the age-standardised rate of change in mortality in the period 2010–17 was also almost two times larger in Saudi Arabia than that in the north Africa and the Middle East GBD region, whereas during the period of 1990–2010 the annual decrease was higher than in Saudi Arabia (–1·69%). The annualised rate of change for global age-standardised mortality was −1·33% for the period 1990–2010, which was slightly lower than the decrease over the period 2010–17 (–1·63%).

**Table:** All-cause mortality estimates for Saudi Arabia, north Africa and the Middle East, and globally in 1990, 2010, and 2017

		**Number of deaths**	**Mortality, per 100 000 population**	**Age-standardised mortality, per 100 000 population**
**Saudi Arabia**				
Year				
	1990	81 249 (72 015–91 473)	495·85 (439·47–558·21)	832·83 (743·47–936·51)
	2010	88 009 (77 171–90 752)	299·46 (275·09–323·50)	740·80 (684·43–789·93)
	2017	94 588 (85 535–105 514)	274·61 (248·33–306·34)	634·90 (595·69–694·39)
Annualised rate of change				
	1990–2010	..	−2·52%	−0·58%
	2010–17	..	−1·23%	−2·20%
**North Africa and Middle East**				
Year				
	1990	2 407 387 (2 369 043–2 447 177)	706·18 (694·93–717·85)	1068·66 (1053·22–1084·80)
	2010	2 589 627 (2 541 104–2 639 784)	490·55 (481·36–500·05)	761·16 (748·57–774·73)
	2017	2 863 213 (2 787 070–2 941 202)	477·06 (464·37–490·05)	695·16 (678·06–713·16)
Annualised rate of change				
	1990–2010	..	−1·82%	−1·69%
	2010–17	..	−0·39%	−1·29%
**Global**				
Year				
	1990	46 478 314 (46 192 588–46 786 253)	861·55 (585·26–867·26)	1079·84 (1072·84–1085·68)
	2010	52 838 311 (52 487 892–53 234 461)	751·3 (746·32–756·93)	827·14 (821·83–833·10)
	2017	55 945 729 (55 356 403–56 516 734)	732·23 (724·52–739·7)	737·73 (729·88–745·35)
Annualised rate of change				
	1990–2010	..	−0·68%	−1·33%
	2010–17	..	−0·36%	−1·63%

Data are for all ages, unless otherwise specified, and data in parentheses are 95% uncertainty intervals.

**Figure 1:**
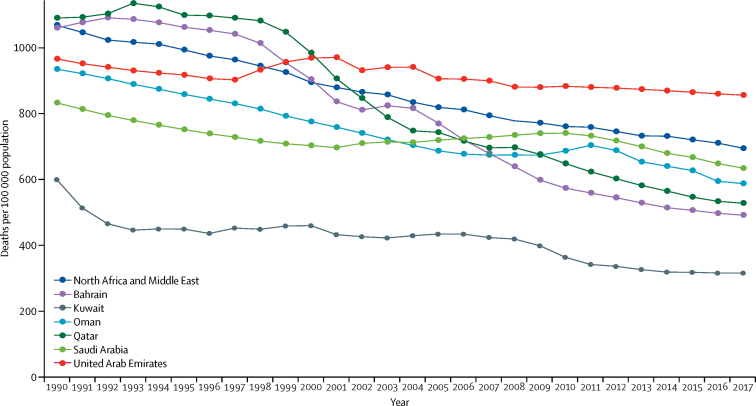
All-cause age-standardised mortality in Saudi Arabia, the other Gulf Cooperative Council Countries, and the north Africa and the Middle East GBD region, for both sexes, 1990–2017 The Gulf Cooperative Council countries are Saudi Arabia, Bahrain, Kuwait, Oman, Qatar, and the United Arab Emirates. GBD=Global Burden of Diseases, Injuries and Risk Factors study.

All-cause age-standardised mortality in Saudi Arabia decreased until 2001, then increased slightly until 2010, thereafter decreasing again until 2017 (figure 1). Compared with the rest of the Gulf Cooperation Council countries, Saudi Arabia's all-cause age-standardised mortality in the 1990s was only higher than that of Kuwait and was lower than that of the other countries.

**Figure 2:**
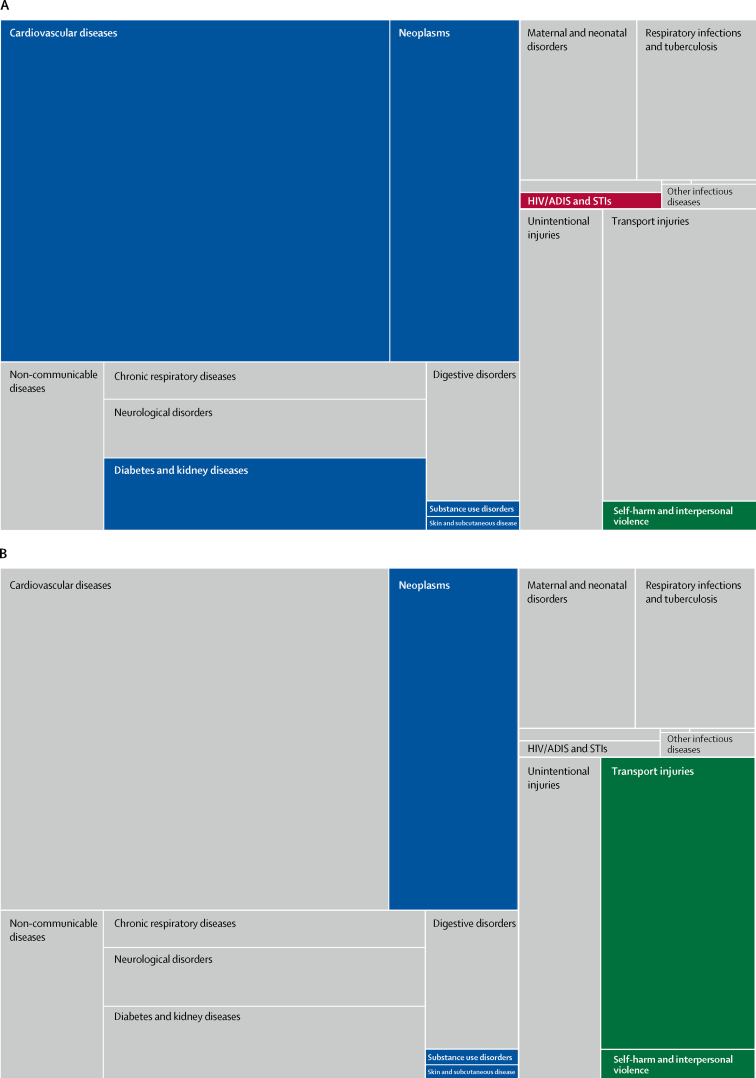
Proportional distribution of causes of death for 1990–2010 (A) and 2010–17 (B) in Saudi Arabia for all Level 2 GBD causes of death Causes of death that increased their proportional burden for the time period are coloured: blue rectangles are non-communicable disease; red rectangles are communicable, maternal, neonatal, and nutritional diseases; and green rectangles are injuries. The causes that remained stable or decreased are in grey. For more detail see the data visualisation tool. GBD=Global Burden of Diseases, Risk factors, and Injuries study. STIs=sexually transmitted infections.

For specific Level 2 causes of all-age mortality, the proportional distribution of deaths due to cardiovascular diseases, neoplasms, HIV/AIDS and sexually transmitted infections, diabetes and kidney diseases, self-harm and interpersonal violence, substance use disorders, and skin and subcutaneous diseases, increased from 1990 to 2010 (figure 2). However, after 2010, many of the above-mentioned causes remained almost stable or decreased, and the annualised rate of change in mortality increased for other causes, including neoplasms, substance use disorders, skin and subcutaneous diseases, and transport injuries (figure 2; more detail is available via the data visualisation tool). The annualised rate of change for non-communicable diseases was −5·35% for 2010–17. Among non-communicable diseases, during the same period of time, the Level 3 cause respiratory infections and tuberculosis had an annualised rate of change of −2·92%.

**Figure 3:**
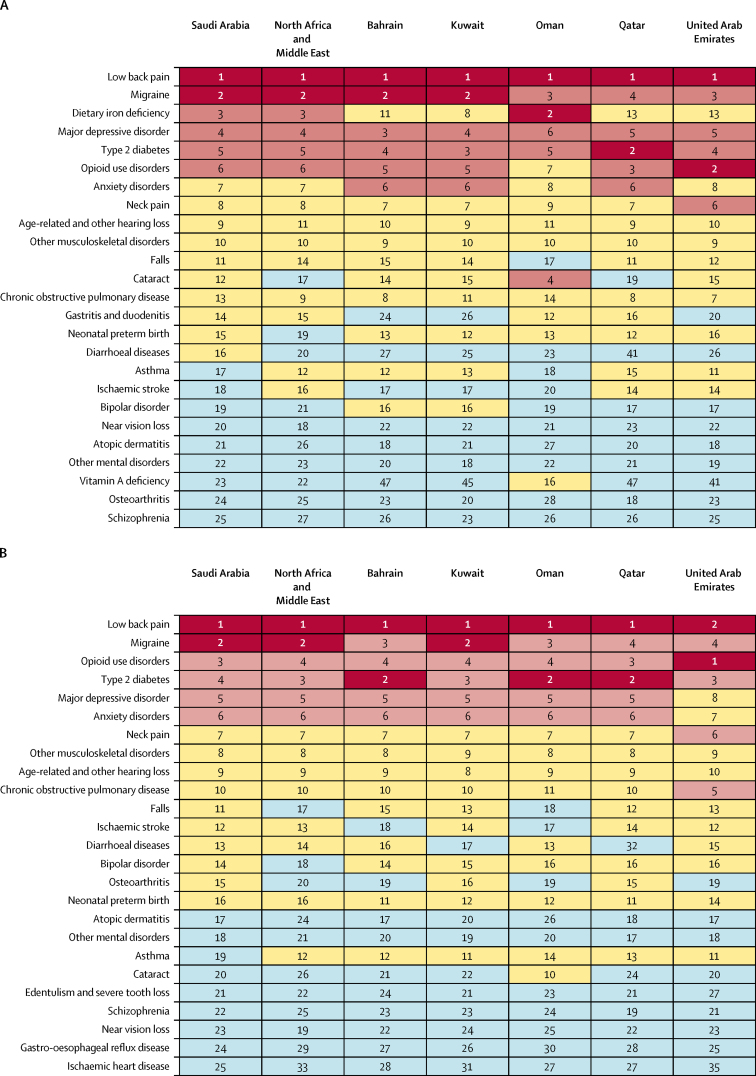
Top-ranked Level 4 causes of age-standardised years lived with disability in both sexes in Saudi Arabia, the other Gulf Cooperation Council countries, and the north Africa and the Middle East GBD region for 1990 (A) and 2017 (B) Numbers are rankings, with colours indicating the scale from low (blue: below 17th) to high (red: first and second) rankings. For more detail see the data visualisation tool. GBD=Global Burden of Diseases, Injuries, and Risk Factors study.

In Saudi Arabia, trends in non-fatal health loss were less pronounced than those of mortality; however, a consistent pattern for (Level 4) causes of YLDs was seen across Saudi Arabia, the Gulf Cooperation Council countries, and the north Africa and the Middle East region. Low back pain, migraine, type 2 diabetes, anxiety disorders, major depressive disorder, and opioid use disorders were the top six causes of YLDs in all areas for 1990, 2010, and 2017, whereas dietary iron deficiency dropped in rank in Saudi Arabia from third in 1990 to below 25th in 2017 (figure 3).

Focusing on Saudi Arabia between 1990, 2010, and 2017, the age-standardised percentage of YLDs (Level 2 and 3 analysis) due to mental disorders (ie, depression and anxiety), substance use disorders (ie, drug and alcohol use disorders), neurological disorders, and neoplasms continued to increase, with a positive annualised rate of change (1990–2017: percentage of YLDs due to mental disorders was 15·6% [95% UI 13·4 to 17·5], substance use disorders was 6·34% [4·73 to 8·16], neurological disorders was 10·4% [7·96 to 13·8], and neoplasms was 0·68% [0·59 to 0·79]). Analysis of the overall burden of all-cause age-standardised YLDs for both sexes, from 1990 to 2017, showed a small change of −7·15% (1990: 11 762 [8774 to 15 186] YLDs per 100 000; 2017: 10 921 [8204 to 14 035] YLDs per 100 000; more detail is available via the data visualisation tool). Our analysis stratified by age group for Saudi Arabia between 1990, 2010, and 2017 showed increasing trends in all-cause YLDs among younger people (15–49 years) and decreasing trends in all-cause YLDs among older people (50–69 years; appendix pp 2–3).

**Figure 4:**
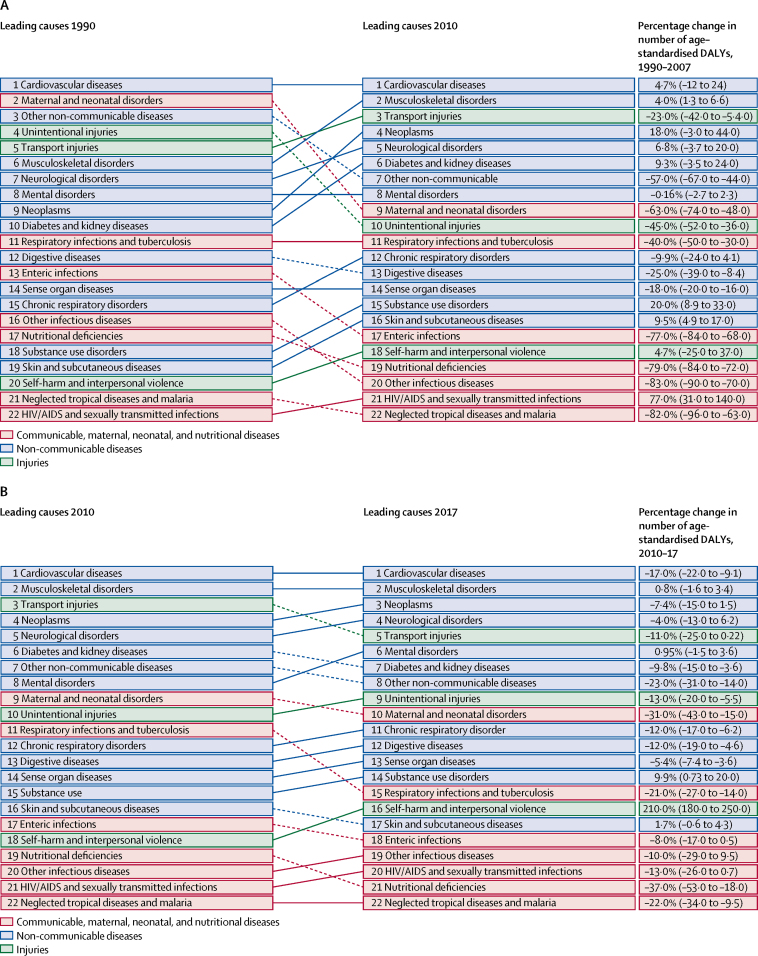
Top 25 Level 2 causes of DALYs in Saudi Arabia for the periods 1990–2010 (A) and 2010–17 (B), both sexes Solid lines indicate increases and dashed lines indicate decreases in rank between periods. Significant changes are shown in bold. For more detail see the data visualisation tool. DALYs=disability-adjusted life-years

Regarding DALY patterns in Saudi Arabia during 1990, 2010, and 2017, cardiovascular diseases remained the top ranked Level 2 cause of DALYs. DALYs due to maternal and neonatal disorders were ranked second in the 1990s but decreased to ninth in 2010, and other non-communicable diseases was ranked third in 1990 and decreased to seventh in 2010 (figure 4). In 2010, musculoskeletal disorders were ranked second and transport injuries were ranked third highest causes of DALYs, followed by neoplasms. From 2010 to 2017, musculoskeletal disorders remained second highest cause while transport injuries decreased to fifth and neoplasms increased to third highest cause of DALYs (figure 4B).

Behavioural and metabolic risk factors (Level 1 analysis) accounted for most attributable all-cause, age-standardised YLDs in Saudi Arabia between 1990 and 2017. In 1990, 10·72% of YLDs (95% UI 9·63–11·91) were attributed to metabolic risk factors, 18·37% (17·03–19·05) to behavioural risks, and 6·75% (6·15–7·38) to environmental and occupational risks (appendix pp 4–6). From 2010 to 2017, the effect of risk factors on YLDs remained in a similar range for Saudi Arabia (2010: metabolic risks: 13·54% [12·18–14·86], behavioural risks: 16·54% [14·91–18·42], environmental and occupational risks: 6·41% [5·62–7·09]; 2017: metabolic risks: 13·3% [11·92–14·63], behavioural risks: 16·62% [14·77–18·58], environmental and occupational risks: 6·21% [5·47–6·95]). Direct comparison of Saudi Arabia with the north Africa and the Middle East GBD region countries between 1990 and 2017 showed that the pattern of YLDs attributable to risk factors in Saudi Arabia was similar to the rest of the countries in the region (appendix pp 4–6). Specifically, in the north Africa and the Middle East region, 1419 (95% UI 1030–1907) YLDs per 100 000 population were attributable to metabolic risks in 2017, an increase compared with 1990. The corresponding 2017 estimate for Saudi Arabia was 1810 (1366–2260) YLDs per 100 000 population attributable to behavioural risks. Burden attributable to behavioural risks steadily decreased in Saudi Arabia from 1990 to 2017, and a similar trend was observed in the north Africa and the Middle East region, with a 9·02% change in YLDs attributable to behavioural risks from 1990 to 2017 (2564 YLDs per 100 000 population in 1990 *vs* 2333 YLDs per 100 000 population in 2017; appendix pp 4–6).

**Figure 5:**
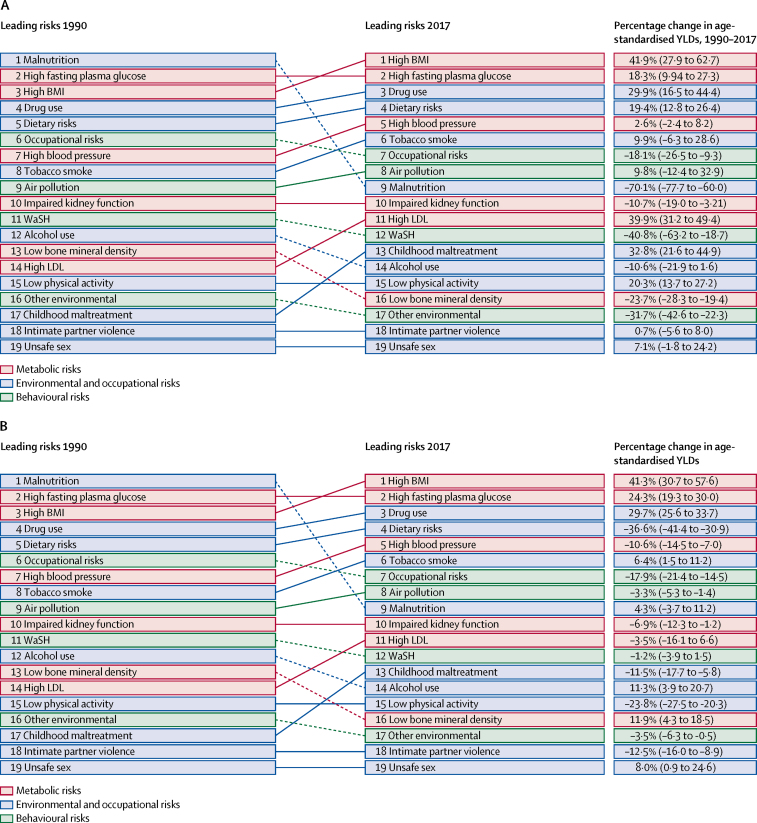
Top 20 Level 2 risk factors for all-cause YLDs in Saudi Arabia (A) and the north Africa and the Middle East GBD region (B) 1990–2017, both sexes Solid lines indicate increases and dashed lines indicate decreases in rank between periods. Significant changes are shown in bold. For more details see the data visualisation tool. BMI=body-mass index. GBD=Global Burden of Diseases, Injuries, and Risk Factors study. WaSH=water, sanitation, and hygiene. YLDs=years lived with disability.

Figure 5 shows trends in all-cause age-standardised YLDs (Level 2 analysis) in Saudi Arabia and the north Africa and the Middle East region due to these factors from 1990 to 2017. Disaggregation of risks (Level 2 analysis) showed a disproportionate increase in YLDs attributable mainly to high BMI, dietary risks, and drug use in Saudi Arabia since the 1990s (figure 5). Burden attributable to tobacco smoke has continuously increased in Saudi Arabia since 1990, with a 9·9% increase in YLDs attributable to tobacco smoke reported for Saudi Arabia from 1990 to 2017 (moved from eighth to sixth rank**]**). Additionally, alcohol use decreased to 14th and dietary risks fourth in Saudi Arabia in 2017. During the same period, YLDs attributable to tobacco smoke increased by 6·4% in the north Africa and the Middle East region (from eighth to sixth), and YLDs due to high BMI increased by 41·3% (ranked first in 2017; figure 5).

In the analysis of YLDs stratified by age group, the relative ranks and attributable YLDs burden due to Level 4 risk factors varied between the three time periods and by age group (appendix pp 7–12). In 1990, for the younger population in Saudi Arabia (aged 15–49 years), the four leading risk factors for YLDs for both sexes combined were, in descending order, drug use, iron deficiency, high BMI, and high fasting plasma glucose concentration. In 2010, YLDs attributable to high BMI increased, moving high BMI to second place and iron deficiency moved down to 11th place. In 2017, the ranking of leading risk factors remained similar to in 2010, but the effects of high BMI, high fasting plasma glucose concentration, high blood pressure, high LDL cholesterol, alcohol use, hypertension, and low physical activity on the YLDs burden increased. These risk-attributable YLDs were associated with various cause groups such as mental disorders, substance use disorders, musculoskeletal disorders, chronic respiratory diseases, cardiovascular diseases, diabetes, urogenital diseases, blood diseases, and endocrine diseases (appendix pp 7–9). Similarly, analysing middle-aged and older Saudis (aged 50–69 years), in 1990, the five leading risk factors for both sexes combined were, in descending order, high BMI, high fasting plasma glucose concentration, high blood pressure, low whole-grain intake, and smoking. In 2010 and 2017, little change in the ranking of risk factors was seen, but their effect on the YLDs burden increased. These risks contributing to YLDs were associated with various cause groups such as neurological disorders, musculoskeletal disorders, chronic respiratory diseases, cardiovascular diseases, neoplasms, diabetes, urogenital diseases, blood diseases, and endocrine diseases (appendix pp 10–12).

Changes in health-care access and quality as measured by the HAQ Index for the period 1990–2016 are more pronounced in Saudi Arabia than in the north Africa and the Middle East GBD region and in most of the Gulf Cooperation Council countries. Although Saudi Arabia's HAQ Index score in 1990 was lowest out of all the regions and countries (49·94, 95% UI 46·96–53·0) alongside the United Arab Emirates (49·81, 43·71–55·41), in 2016, Saudi Arabia had a higher HAQ Index score (77·13, 74·92–79·35) than most of the Gulf Cooperation Council countries (except Qatar and Kuwait) and the north Africa and the Middle East GBD region (appendix p 13).

## Discussion

Age-standardised mortality in Saudi Arabia decreased slowly from 1990 to 2010 (annualised rate of change −0·58%), and this decrease accelerated from 2010 to 2017 (–2·20%). The health-care reform in Saudi Arabia that began in 2010 seems to have increased the pace of Saudi Arabia's population health improvement in comparison with the overall north Africa and the Middle East GBD region. Causes of death that decreased during 2010–17 in Saudi Arabia included cardiovascular diseases, diabetes and kidney diseases, maternal and neonatal disorders, respiratory infections and tuberculosis and nutritional deficiencies. Mortality due to communicable diseases decreased between 2010 and 2017. The disability burden was attributed mainly to cardiovascular disease but also transport injuries (which deceased in 2010–17). Attributable YLDs due to metabolic and behavioural risk factors increased in Saudi Arabia between 1990–2010 and 2010–17 for the population aged 15–49 years, with increases in the burden of YLDs attributable to high BMI, high fasting plasma glucose concentration, and drug use. The trend that risk factors such as high LDL cholesterol, alcohol use, hypertension, and low physical activity continued to increase across the time period was notable. Prevention and management of these metabolic and behavioural risk factors is a major challenge to health policy makers in Saudi Arabia. Our findings will help them to escalate public policy actions on key risk factors in the younger population and highlight the importance of continued health interventions and education.

Our findings that decreases in all-cause age-standardised mortality were faster after 2010 than before 2010 are consistent with those of WHO for non-communicable disease mortality.^23^ WHO's analysis for Saudi Arabia also noted that in 2016 non-communicable diseases accounted for 73% of all deaths (some major causes of death being cardiovascular disease, cancer, chronic respiratory diseases, and diabetes).^24^ We found a similar magnitude of mortality by cause between 2010 and 2017. Analysing specific-causes of mortality in 2010–17, we found that mortality due to specific non-communicable causes such as cardiovascular disease and diabetes and kidney disease were decreased. Despite these decreases, these diseases remained among the top-ranked causes of mortality. Additionally, we found that mortality due to neoplasms, skin and subcutaneous diseases, and substance use in Saudi Arabia were continuing the increase. These increases in mortality due to cancer and skin diseases could be attributed to changing demographic trends in Saudi Arabia, and could be an impetus to the local public health authorities to actively implement healthy ageing strategies.^26^ By contrast, mortality due to communicable diseases continues to be controlled, a result that is in accordance with the previous GBD 2010 Saudi Arabia subanalysis.^9^

The trends of disease burden in Saudi Arabia could be a result of several factors, and the full explanation is most likely multi-dynamic. Although mortality is a robust health outcome, disability burden and its drivers (risk factors) should be analysed in parallel with Saudi Arabia's population structure. First, our analysis reports that between 1990, 2010, and 2017, the all-cause age-standardised YLDs burden for the total population of Saudi Arabia decreased negligibly. Mental disorders, substance use disorders, neurological disorders, and neoplasms were among the causes of YLDs with a positive annual rate of change during this 27-year period. The burden of mental disorders is reported to be high in the Arab region, with social stigma reported in the local society and women having the highest burden.^27^, ^28^, ^29^ Increases in mental disorders^30^, ^31^ and misuse of drugs and medications among the Saudi population^32^, ^33^ support our findings and call for enhancement of preventive and mental health education policies.^11^

Second, additional analyses by age group (15–49 and 50–69 years) showed increasing trends in all-cause YLDs between 2010 and 2017 for the younger population and decreasing trends in middle-aged to older Saudis. This finding is particularly notable, along with the fact that high BMI, tobacco smoke, high fasting plasma glucose concentration, dietary risks, drug use, and alcohol consumption are major contributors to disability in Saudi Arabia. Results from the Saudi Health Interview Survey support high prevalence of these risk factors in the Saudi Arabian population.^8^, ^34^, ^35^, ^36^, ^37^, ^38^ Additionally, despite high prevalence of metabolic risk factors such as hypertension and high LDL cholesterol, a large proportion of the population is undiagnosed or uncontrolled, with a lot of in-country variation.^39^, ^40^ The younger Saudi population tends to be obese and sedentary, and mostly adheres to unhealthy dietary patterns,^8^ whereas older Saudis have a high prevalence of diabetes, abnormal arterial pressure, and musculoskeletal disorders.^32^ The all-cause age-standardised YLDs burden in Saudi Arabia deserves special attention, much like the rest of the Gulf Cooperation Council countries and the north Africa and the Middle East region.^25^ Based on our findings, Saudi Arabia and Gulf Cooperation Council countries have similar patterns of causes of YLDs. In 2017, low back pain, migraines, opioid use disorders, major depressive disorder, type 2 diabetes, and anxiety disorders were major challenges for Saudi Arabia and the rest of the comparator locations. The ranking of disability causes among the Gulf Cooperation Council countries, the north Africa and the Middle East GBD region, and Saudi Arabia showed little discrepancy between 1990 and 2017.

Third, the high prevalence of risk factors among the Saudi Arabian population could be explained by the fact that Saudis tend to rarely seek preventive health examinations and most use health-care services only for treatment.^11^ The rural population has also been reported to use and access primary health-care services less than urban residents do.^10^ Female Saudis also have low rates of breast cancer screening.^41^ Health-care services in Saudi Arabia are freely accessible; however, dissatisfaction among the general public is high and they do not tend to perceive that risk factors are inversely related to their actual health status.^11^ The Saudi Arabian HAQ Index improved between 1990 and 2016 (data for 2017 were not yet available at the time of writing), but users' dissatisfaction, along with the low level of disease awareness among Saudis, remain major barriers. To address these issues, the Saudi Arabian ministry of health urgently needs to understand the determinants of Saudis' low use of and dissatisfaction with preventive health-care services.

Fourth, the Saudi Arabian ministry of health has increased investment in health-care services, recognising major challenges such as accessibility and health personnel development.^4^ Specifically, these actions took place after 2010 and have continued to date. As part of the National Transformation Program 2020 (Vision 2030), the ministry of health announced advanced investments, reformation, and enhancement of the Saudi Arabian health-care system. Relevant strategic objectives of Vision 2030 specifically address risk factors such as BMI and tobacco use (strategic objective number 13). Additional actions started in 2012, including collaboration with international organisations to address the topic of increasing non-communicable diseases and their risk factors.^7^ Some of the effects of these actions are reflected in the improvement of the HAQ Index between 1990 and 2016; however, the high disability burden (eg, increase in musculoskeletal and neurological disorder DALYs from 1990 to 2017) and its risk factors call for increased investment in preventive measures at individual and population levels.^11^ Additionally, curative and secondary preventive care could be enhanced towards cost-effective interventions that would facilitate equity and long-term sustainability of health-care expenditures. Curative, secondary, and tertiary health-care services also need to continue to address the current high burden of risk factors and disability. This high burden of risk factors might be the reason why mortality due to cardiovascular diseases showed a decreasing annualised rate of change from 2010 to 2017 but remained the major cause of mortality in Saudi Arabia. Furthermore, a national chronic disease surveillance system with advanced mapping techniques is urgently needed to track incidence, deaths, and actual information on modifiable risk factors, as is enhancement of primary health-care services. A public health strategy is needed to prevent the increasing burden of non-fatal chronic diseases. Cost-effective primary care interventions need to be developed at the community level to reduce chronic disease incidence, which would need to be supported by a strong health surveillance system. Since the beginning of the 20th century, the Directorate of Public Health in Saudi Arabia has identified a lack of health awareness and education as a priority issue.^42^ Since then, health education activities have increased, focused on communicable and chronic diseases,^42^ but population health awareness still remains low.^43^ Population health education and awareness strategies could establish effective means to manage the disability burden and its related risk factors.^34^, ^37^, ^44^ The combined mortality and disability burden due to transportation injuries remains an important contributors to the total burden, although it was flagged as a key risk factor since 2015.^9^, ^45^ Population driving education programmes^45^ should be part of these strategies in parallel with advanced control systems (ie, traffic-control legislation).^7^

Finally, to better understand Saudi Arabia's population disease and disability burden and the potential role of the health-care system, the country's current and future economic development and environmental sustainability planning must be further analysed. Saudi Arabia's economy is dependent on oil revenue,^46^ and in the past few years new economic plans have been announced to transform the economy into a modern industrial one that would be less oil dependent. Some of the plans for socioeconomic diversification have already been achieved (ie, social development) and others are progressing at a slower pace (ie, foreign investments, privatisation).^47^ However, Saudi Arabia's economy is still largely dependent on oil resources, which is linked with a large carbon footprint and environmental pollution. Data have shown that between 1970 and 2012, carbon dioxide emissions increased almost eight times in Saudi Arabia, whereas the global carbon footprint increased by 1·3 times.^48^ Data have shown that if Saudi Arabia could focus its economy on gas consumption, it would reduce the region's total carbon footprint.^46^ Along with these issues, Saudi Arabia's electricity consumption is one of the highest in the world, and it uses almost double the water that other countries use on average.^48^ Renewable energy sources have been proposed as one measure to address these challenges;^49^ however, enhancement in specific environmental sustainability policies is needed. Based on our data analysis, air pollution, unsafe water, and other environmental factors remain in the top 17 health risks for Saudi Arabia in 2017. Taking all these suggestions into account, a modernised health-care system linked with efficient environmental sustainability policies could ensure the environmental protection of the area along with a better population quality of life in the future.

This study has several strengths and limitations. This study is among the first to use the GBD 1990–2017 data analysing the health status of Saudi Arabia after the major health-care investments and actions in the country in 2010. The present analysis shares the limitations of the GBD 2017 study, including the challenges of capturing all sources of uncertainty, lags in data availability, variation in coding practices, and other biases, and limitations of existing analytical tools, which might not fully capture temporal trends in mortality, incidence, and prevalence in Saudi Arabia and in the north Africa and the Middle East region (where national health statistics are not collected rigorously). For 1990–2017, available data sources for the adult population of Saudi Arabia included surveys (most recently done in 2006), census data that are adjusted (in 2004) and unadjusted (2016), WHO vital registration systems (most recently updated in 2009), and UN population estimates and projections (updated in 2017) as additional comparative sources. Data from new population surveys done by the Saudi Arabian ministry of health (such as the Saudi Health Interview Survey, started in 2012) will further help in data completeness among fatal and non-fatal health outcomes. Additionally, the HAQ Index analysis was limited to using data from 1990 to 2016. Furthermore, the missing health information regarding Saudi Arabian expatriates' data in the GBD dataset might bias the interpretation of the presented findings. Also, in a continuously developing country like Saudi Arabia, improved quality of and access to health care over the years might have improved the detection of risk factors and disease burden, which could result in some of the reported increases in non-communicable disease rates. The GBD methodology does not account for both qualitative cultural differences and differential access to resources in different countries. For this reason, disease and disability burden comparisons between Saudi Arabia, the north Africa and the Middle East region, and Gulf Cooperation Council countries also share this limitation. Although some of these countries share religion and similar ethnicity, differences exist among them that have a bearing on population health. The north Africa and the Middle East region is a heterogeneous group of various different country-income-level countries, some of which are well known for the long-term armed conflict taking place. Thus, the comparisons in this Article should be interpreted with caution.

Comparing the periods 1990–2010 and 2010–17, we found evidence of decreased all-cause age-standardised mortality in Saudi Arabia. Decreased mortality was seen in countries in the Gulf Cooperation Council and in the north Africa and the Middle East region for the same periods; however, specific chronic disease burden, such as mental disorders, substance use disorders, and neurological disorders, and risk factors, such as high BMI, was reported at high rates across all age groups in the Saudi Arabian population. Despite the increase in HAQ Index level, improvement in non-fatal health outcomes in Saudi Arabia has been lacking especially after 2010. All these aspects call for implementation of programmes targeting health and lifestyle change education in young age groups, and enhancement of education and awareness activities. In adults in Saudi Arabia, community and primary care interventions focused on metabolic and behavioural health risk factors could be two major aspects of health policy planning. Finally, the development of a modernised health-care system linked with environmentally sustainable policies seems to be the key for future environmental protection and a healthier population.

Correspondence to: Dr Stefanos Tyrovolas, Department of Nutrition Science and Dietetics, School of Health Science and Education, Harokopio University, Athens 17671, Greece s.tyrovolas@pssjd.org

## Data sharing

To download the data used in these analyses, please visit the Global Health Data Exchange.
