# Supplementary material for: The burden of disease in Saudi Arabia 1990–2017: results from the Global Burden of Disease Study 2017
Source: Lancet Planet Health. 2020 May 19;4(5):e195–208. doi: 10.1016/S2542-5196(20)30075-9 (PMC7653403; doi:10.1016/S2542-5196(20)30075-9)
Supplement: Supplementary appendix [file mmc1.pdf]

THE LANCET
Planetary Health

Supplementary appendix

This appendix formed part of the original submission and has been peer reviewed. We post it as supplied by the authors.

Supplement to: GBD 2017 Saudi Arabia Collaborators. The burden of disease in Saudi Arabia 1990–2017: results from the Global Burden of Disease Study 2017. *Lancet Planet Health* 2020: **4:** e195–208.

The burden of disease in Saudi Arabia 1990–2017: results from the Global Burden of Disease Study 2017

Table of Contents

Supplementary Figure 1. All-cause YLDs rates at age groups 15–49 and 50–69 years old in both sexes, in Saudi Arabia, between 1990 and 2017……………………………………….....2
Supplementary Figure 2. Percentage of all cause, age-standardised YLDs attributed to metabolic, behavioural, and environmental risks (Level 1 analysis), in both sexes, projected in North Africa and Middle East countries in 1990, 2010, and 2017 ……………………………………………………………………………………………4
Supplementary Figure 3. YLD rates attributed to metabolic, behavioural, and environmental risks (Level 4 analysis), in both sexes, for the age group 15–49 years old, in Saudi Arabia, 1990, 2010, and 2017……………….…………………………………………………………………...7
Supplementary Figure 4. YLDs rates attributed to metabolic, behavioural, and environmental risks (Level 4 analysis), in both sexes, for the age group 50–69 years old, in Saudi Arabia, 1990, 2010, and 2017…………………………………………………………………………………......10
Supplementary Figure 5. Healthcare Access and Quality (HAQ) Index rates, for both sexes, in Saudi Arabia, Gulf Council Countries, and the North Africa and Middle East region during 1990–2016…………………………………………………………………………….....13

Supplementary Figure 1. All-cause YLDs rates at age groups 15–49 and 50–69 years old in both sexes, in Saudi Arabia, between 1990 and 2017

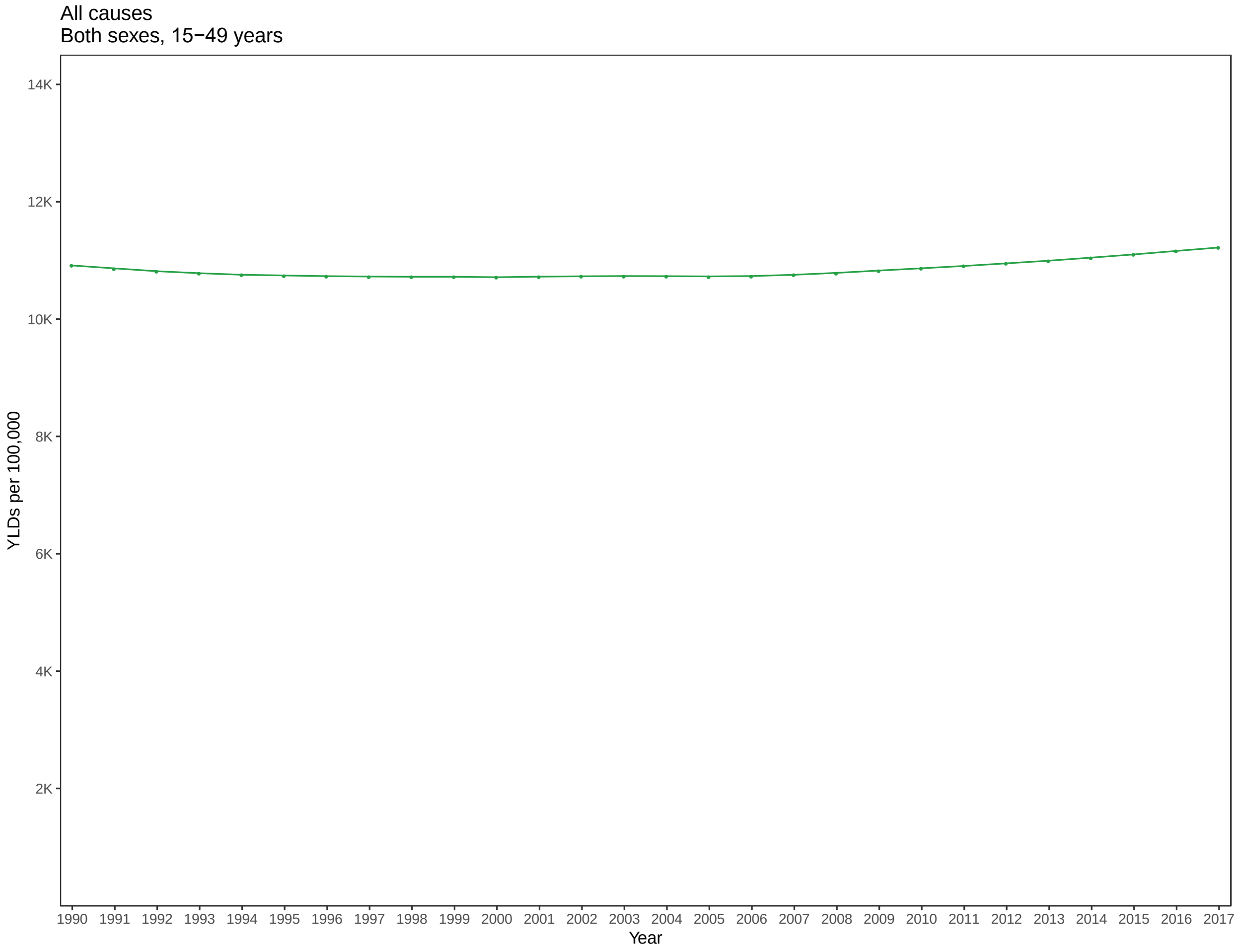

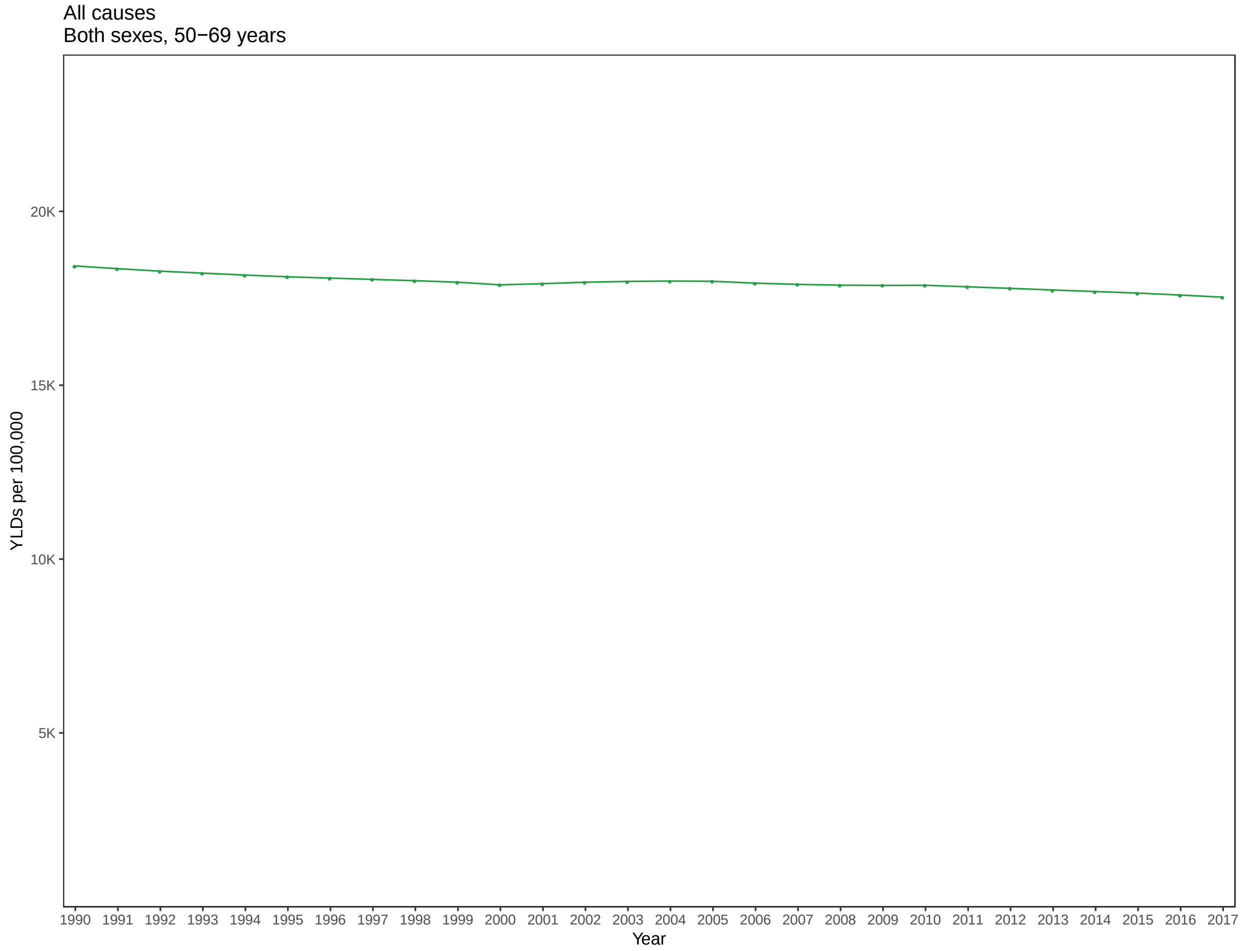

YLDs: Years lived with disability

Supplementary Figure 2. Percentage of all cause, age-standardised YLDs attributed to metabolic, behavioural, and environmental risks (Level 1 analysis), in both sexes, projected in North Africa and Middle East countries in 1990, 2010, and 2017

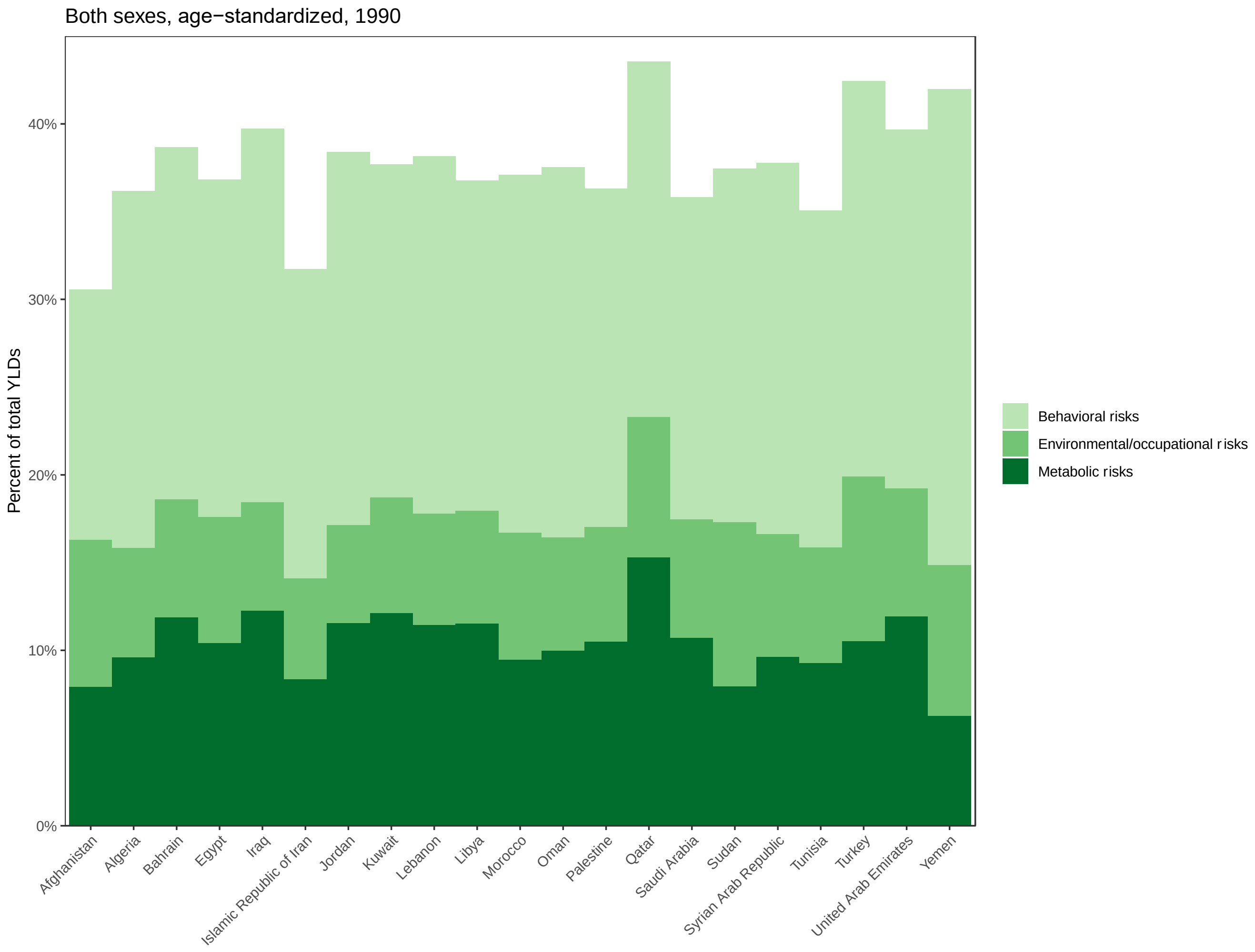

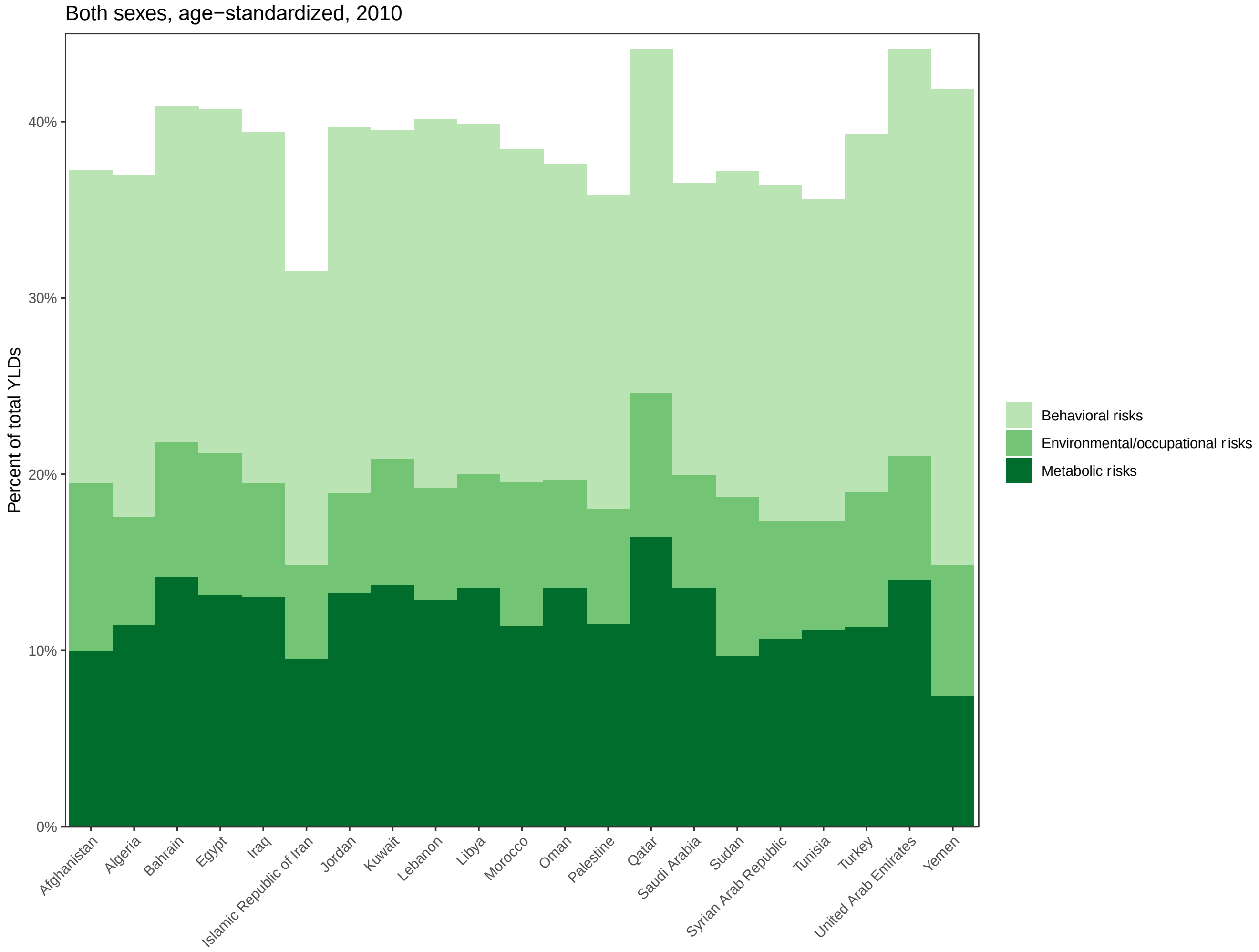
Both sexes, age−standardized, 2010
Percent of total YLDs
0%
10%
20%
30%
40%
Afghanistan
Algeria
Bahrain
Egypt
Iraq
Islamic Republic of Iran
Jordan
Kuwait
Lebanon
Libya
Morocco
Oman
Palestine
Qatar
Saudi Arabia
Sudan
Syrian Arab Republic
Tunisia
Turkey
United Arab Emirates
Yemen
Behavioral risks
Environmental/occupational risks
Metabolic risks

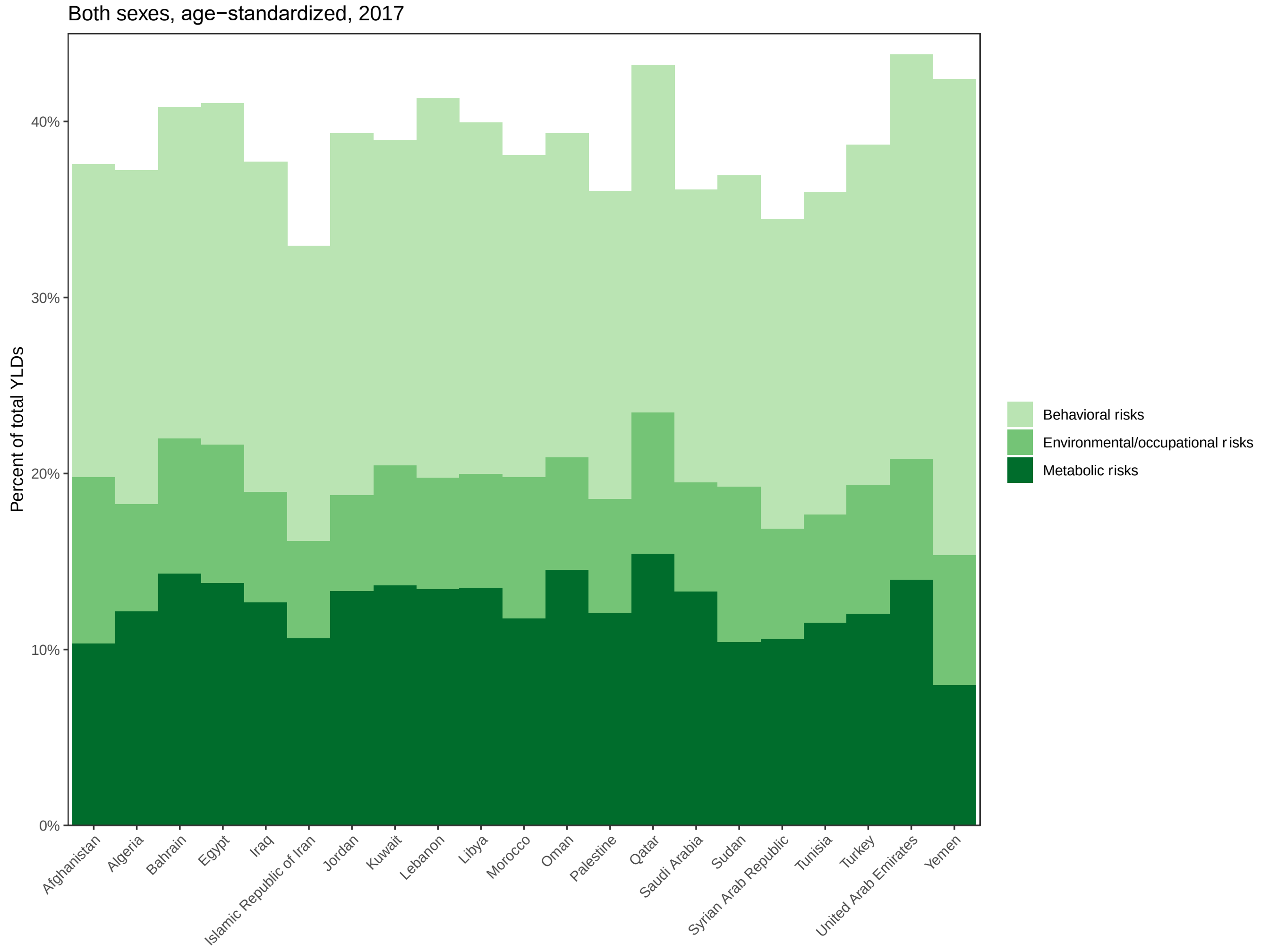

YLDs: Years lived with disability

Supplementary Figure 3. YLD rates attributed to metabolic, behavioural, and environmental risks (Level 4 analysis), in both sexes, for the age group 15–49 years old, in Saudi Arabia, 1990, 2010, and 2017

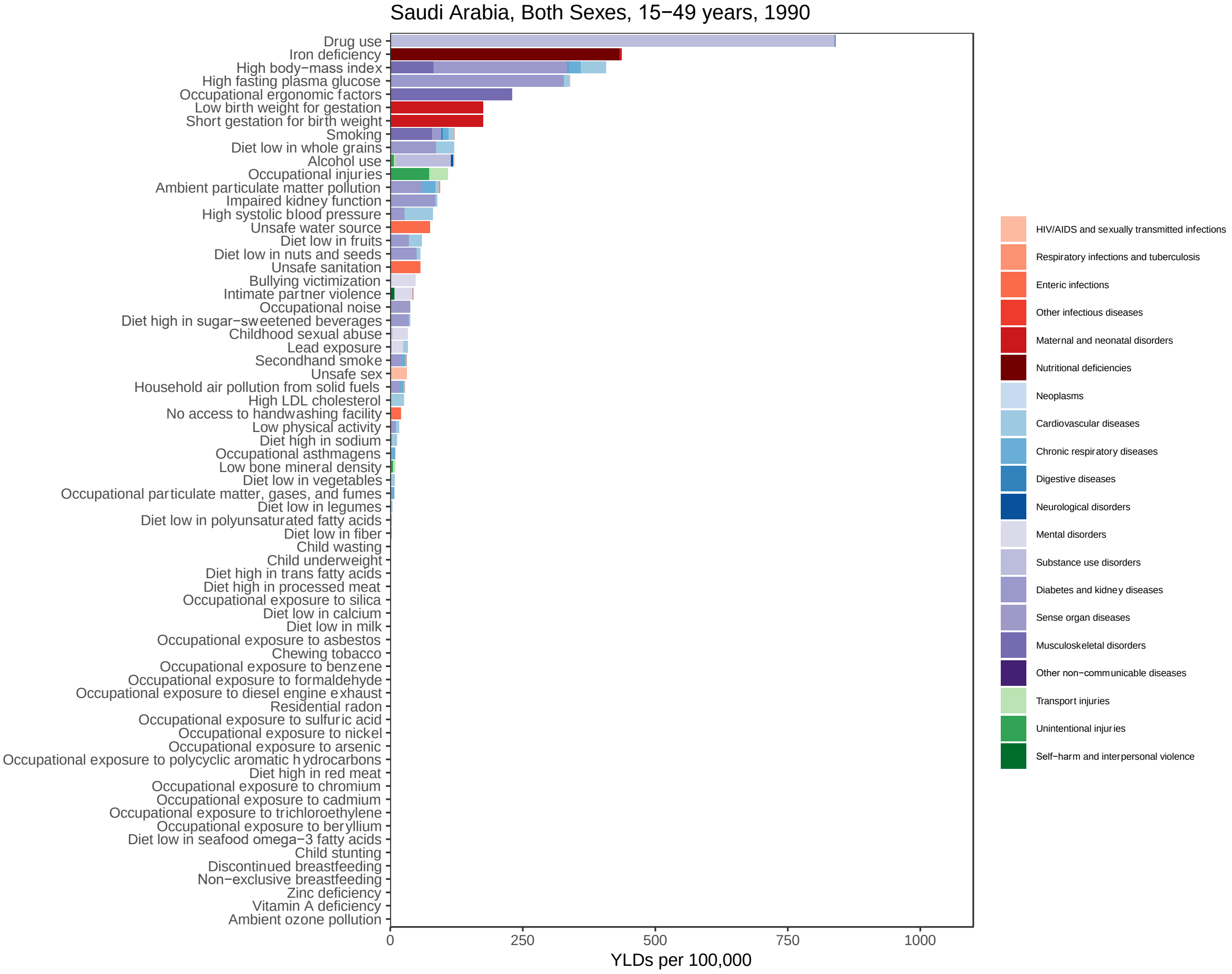

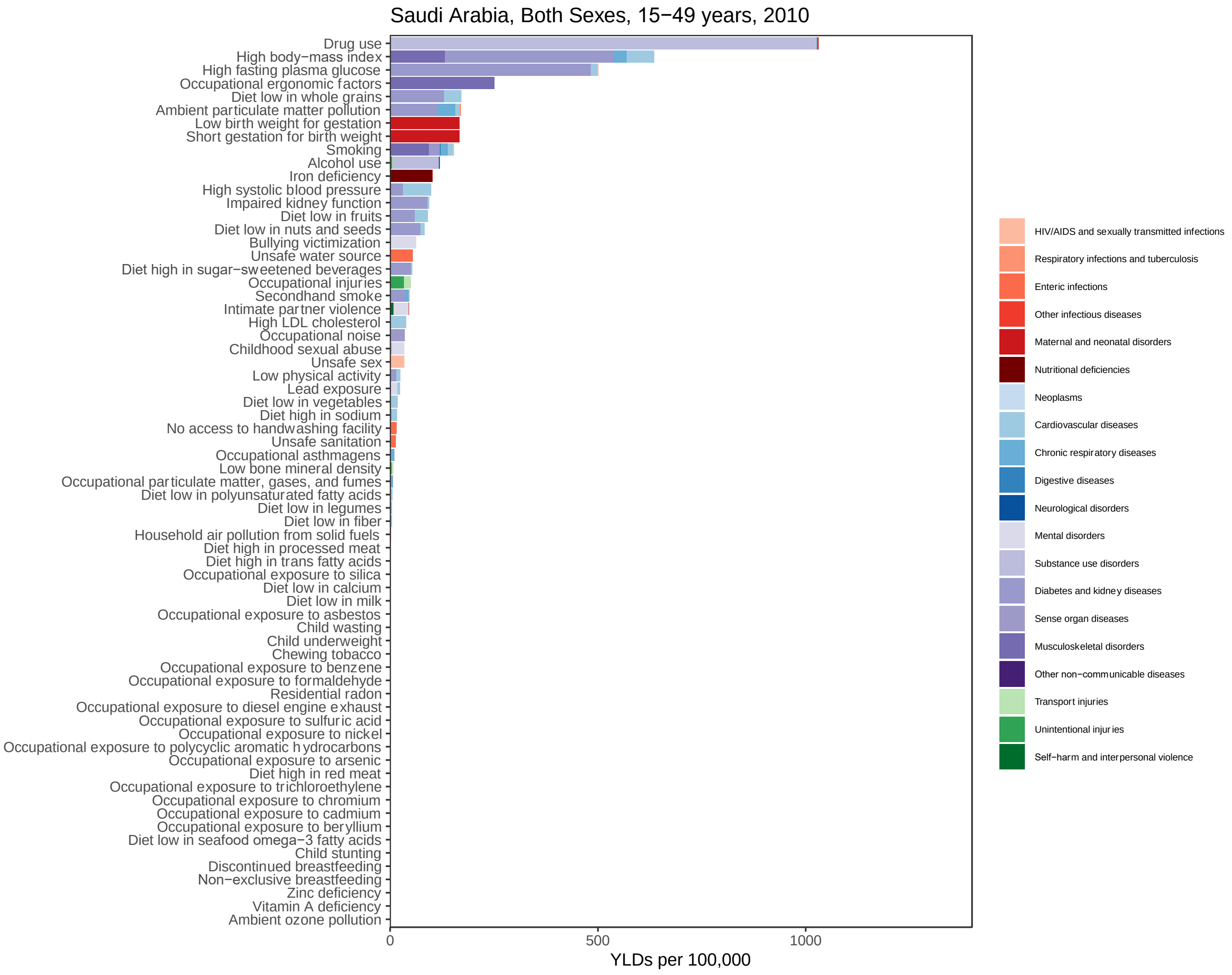
Saudi Arabia, Both Sexes, 15−49 years, 2010
Drug use
High body−mass index
High fasting plasma glucose
Occupational ergonomic factors
Diet low in whole grains
Ambient particulate matter pollution
Low birth weight for gestation
Short gestation for birth weight
Smoking
Alcohol use
Iron deficiency
High systolic blood pressure
Impaired kidney function
Diet low in fruits
Diet low in nuts and seeds
Bullying victimization
Unsafe water source
Diet high in sugar−sweetened beverages
Occupational injuries
Secondhand smoke
Intimate partner violence
High LDL cholesterol
Occupational noise
Childhood sexual abuse
Unsafe sex
Low physical activity
Lead exposure
Diet low in vegetables
Diet high in sodium
No access to handwashing facility
Unsafe sanitation
Occupational asthmagens
Low bone mineral density
Occupational particulate matter, gases, and fumes
Diet low in polyunsaturated fatty acids
Diet low in legumes
Diet low in fiber
Household air pollution from solid fuels
Diet high in processed meat
Diet high in trans fatty acids
Occupational exposure to silica
Diet low in calcium
Diet low in milk
Occupational exposure to asbestos
Child wasting
Child underweight
Chewing tobacco
Occupational exposure to benzene
Occupational exposure to formaldehyde
Residential radon
Occupational exposure to diesel engine exhaust
Occupational exposure to sulfuric acid
Occupational exposure to nickel
Occupational exposure to polycyclic aromatic hydrocarbons
Occupational exposure to arsenic
Diet high in red meat
Occupational exposure to trichloroethylene
Occupational exposure to chromium
Occupational exposure to cadmium
Occupational exposure to beryllium
Diet low in seafood omega−3 fatty acids
Child stunting
Discontinued breastfeeding
Non−exclusive breastfeeding
Zinc deficiency
Vitamin A deficiency
Ambient ozone pollution
0
500
1000
YLDs per 100,000
HIV/AIDS and sexually transmitted infections
Respiratory infections and tuberculosis
Enteric infections
Other infectious diseases
Maternal and neonatal disorders
Nutritional deficiencies
Neoplasms
Cardiovascular diseases
Chronic respiratory diseases
Digestive diseases
Neurological disorders
Mental disorders
Substance use disorders
Diabetes and kidney diseases
Sense organ diseases
Musculoskeletal disorders
Other non−communicable diseases
Transport injuries
Unintentional injuries
Self−harm and interpersonal violence

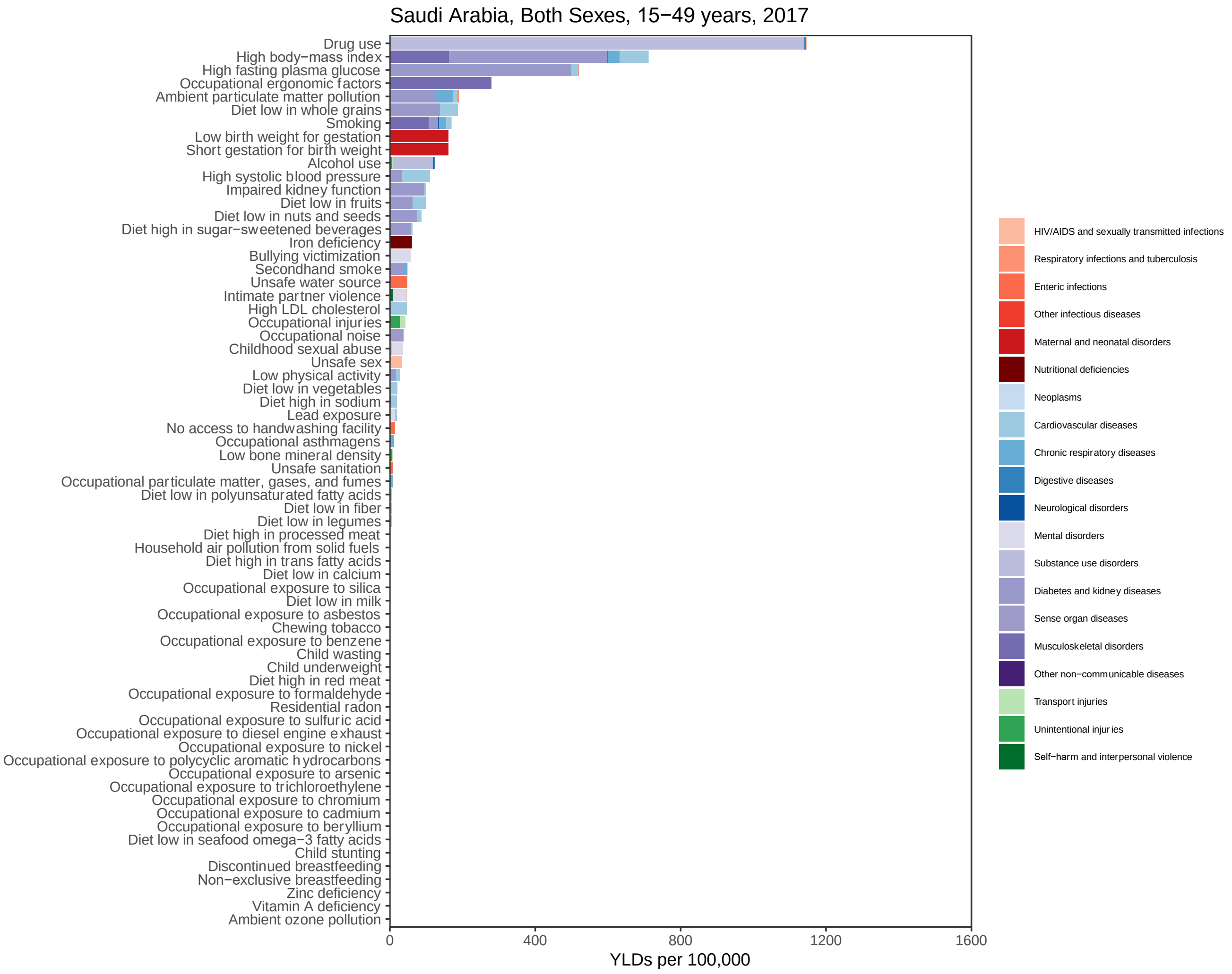

YLDs: Years lived with disability

Supplementary Figure 4. YLDs rates attributed to metabolic, behavioural, and environmental risks (Level 4 analysis), in both sexes, for the age group 50–69 years old, in Saudi Arabia, 1990, 2010, and 2017

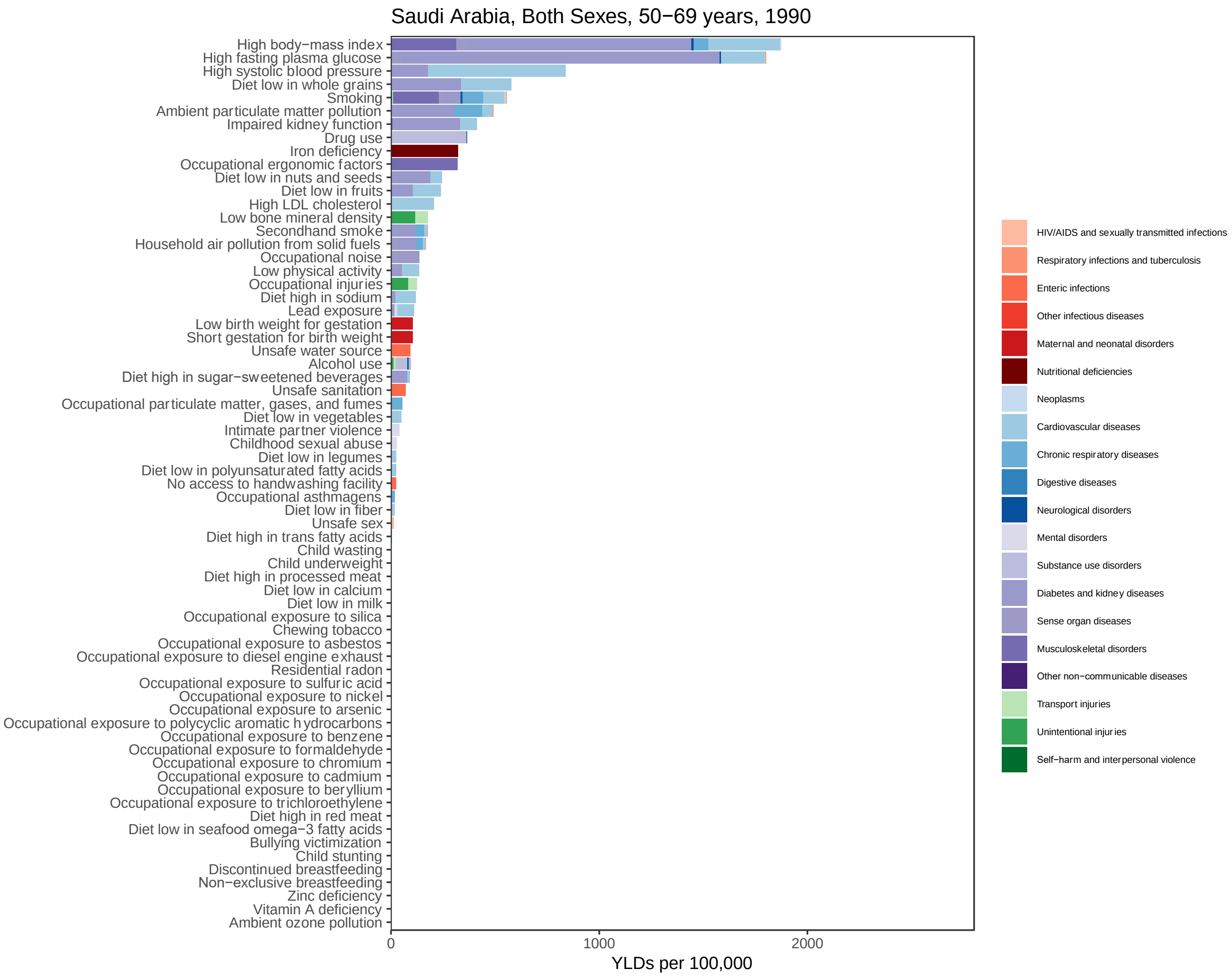

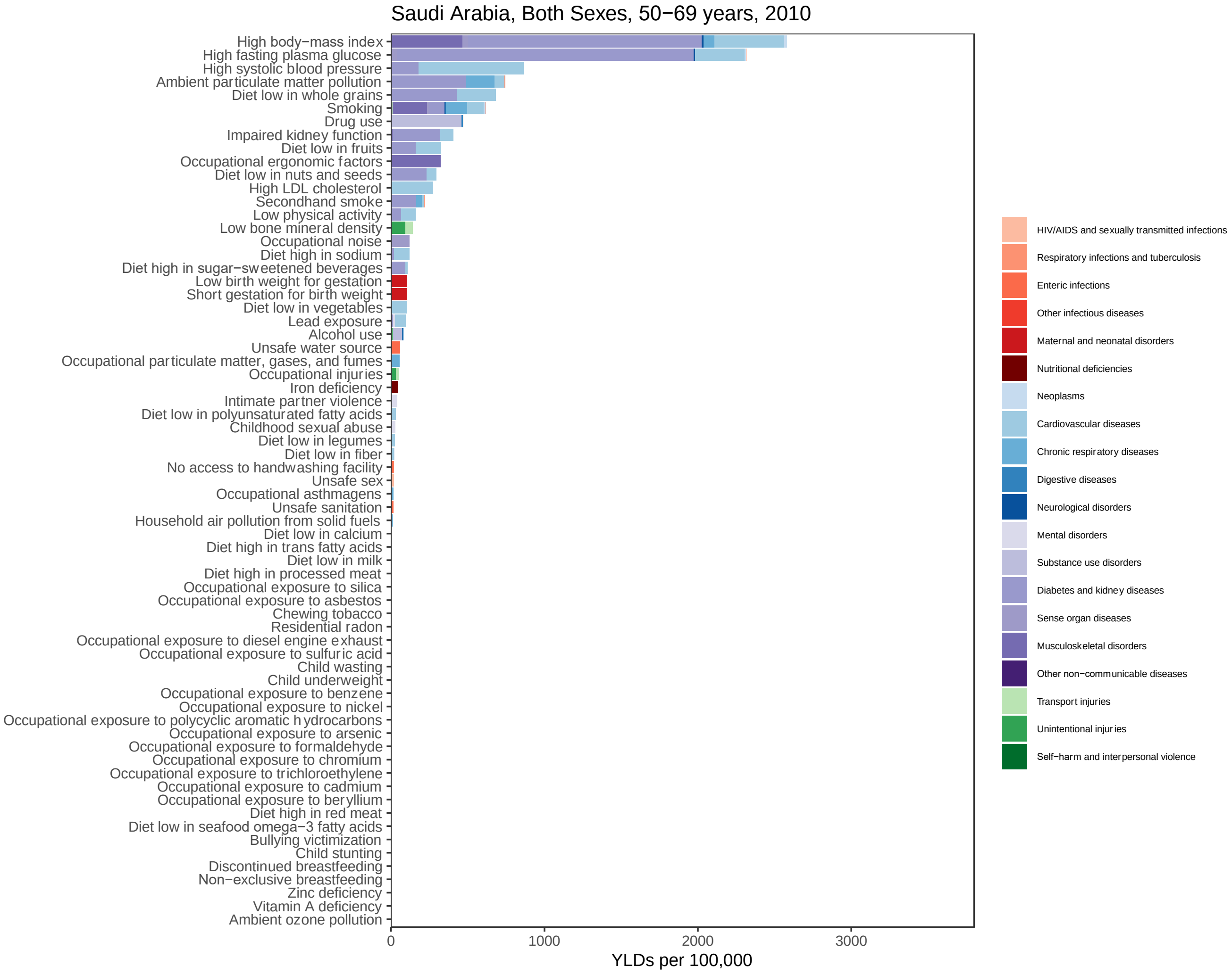
Saudi Arabia, Both Sexes, 50−69 years, 2010
High body−mass index
High fasting plasma glucose
High systolic blood pressure
Ambient particulate matter pollution
Diet low in whole grains
Smoking
Drug use
Impaired kidney function
Diet low in fruits
Occupational ergonomic factors
Diet low in nuts and seeds
High LDL cholesterol
Secondhand smoke
Low physical activity
Low bone mineral density
Occupational noise
Diet high in sodium
Diet high in sugar−sweetened beverages
Low birth weight for gestation
Short gestation for birth weight
Diet low in vegetables
Lead exposure
Alcohol use
Unsafe water source
Occupational particulate matter, gases, and fumes
Occupational injuries
Iron deficiency
Intimate partner violence
Diet low in polyunsaturated fatty acids
Childhood sexual abuse
Diet low in legumes
Diet low in fiber
No access to handwashing facility
Unsafe sex
Occupational asthmagens
Unsafe sanitation
Household air pollution from solid fuels
Diet low in calcium
Diet high in trans fatty acids
Diet low in milk
Diet high in processed meat
Occupational exposure to silica
Occupational exposure to asbestos
Chewing tobacco
Residential radon
Occupational exposure to diesel engine exhaust
Occupational exposure to sulfuric acid
Child wasting
Child underweight
Occupational exposure to benzene
Occupational exposure to nickel
Occupational exposure to polycyclic aromatic hydrocarbons
Occupational exposure to arsenic
Occupational exposure to formaldehyde
Occupational exposure to chromium
Occupational exposure to trichloroethylene
Occupational exposure to cadmium
Occupational exposure to beryllium
Diet high in red meat
Diet low in seafood omega−3 fatty acids
Bullying victimization
Child stunting
Discontinued breastfeeding
Non−exclusive breastfeeding
Zinc deficiency
Vitamin A deficiency
Ambient ozone pollution
0
1000
2000
3000
YLDs per 100,000
HIV/AIDS and sexually transmitted infections
Respiratory infections and tuberculosis
Enteric infections
Other infectious diseases
Maternal and neonatal disorders
Nutritional deficiencies
Neoplasms
Cardiovascular diseases
Chronic respiratory diseases
Digestive diseases
Neurological disorders
Mental disorders
Substance use disorders
Diabetes and kidney diseases
Sense organ diseases
Musculoskeletal disorders
Other non−communicable diseases
Transport injuries
Unintentional injuries
Self−harm and interpersonal violence

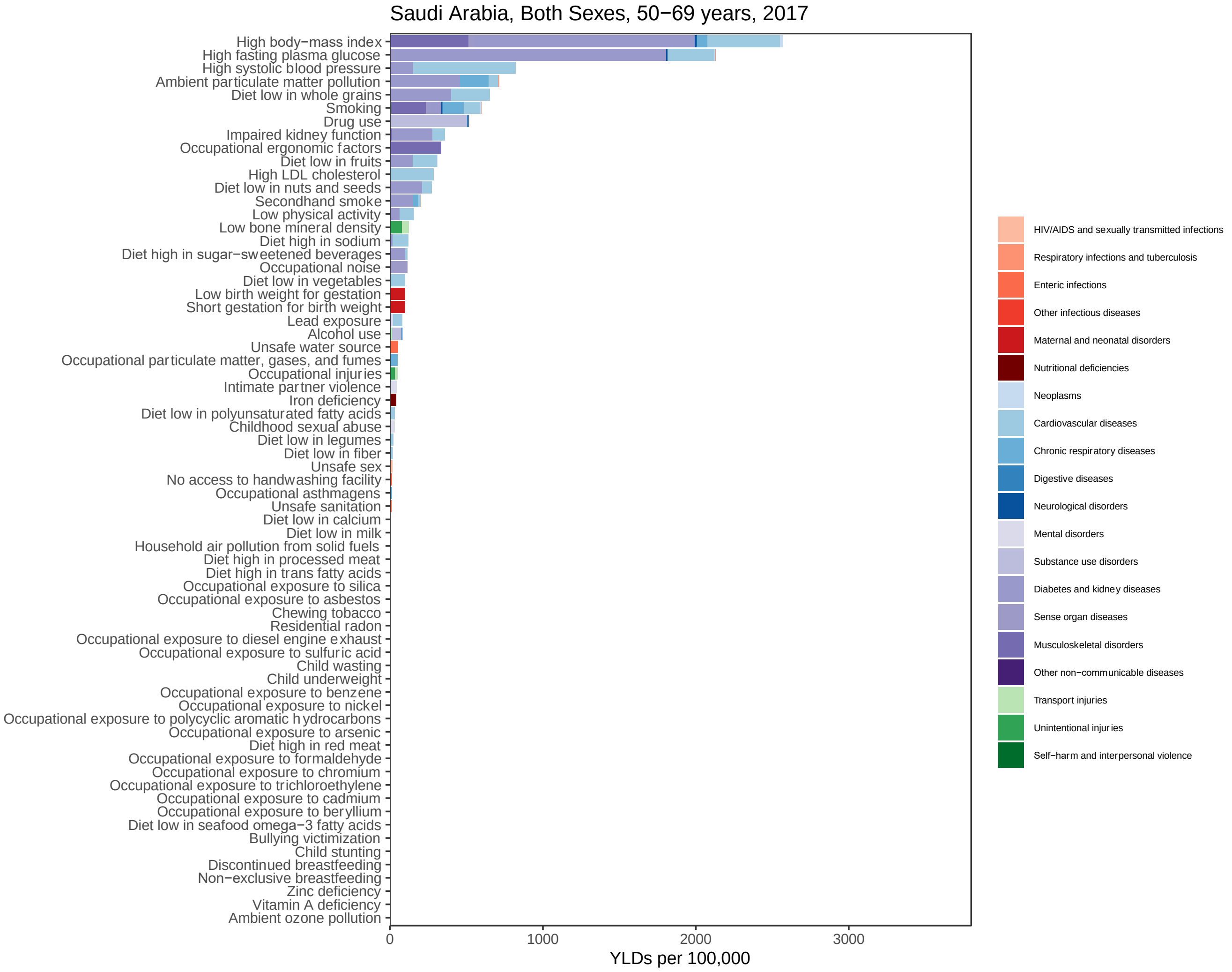

YLD: Years lived with disability

Supplementary Figure 5. Healthcare Access and Quality (HAQ) Index rates, for both sexes, in Saudi Arabia, Gulf Council Countries, and the North Africa and Middle East region during 1990–2016

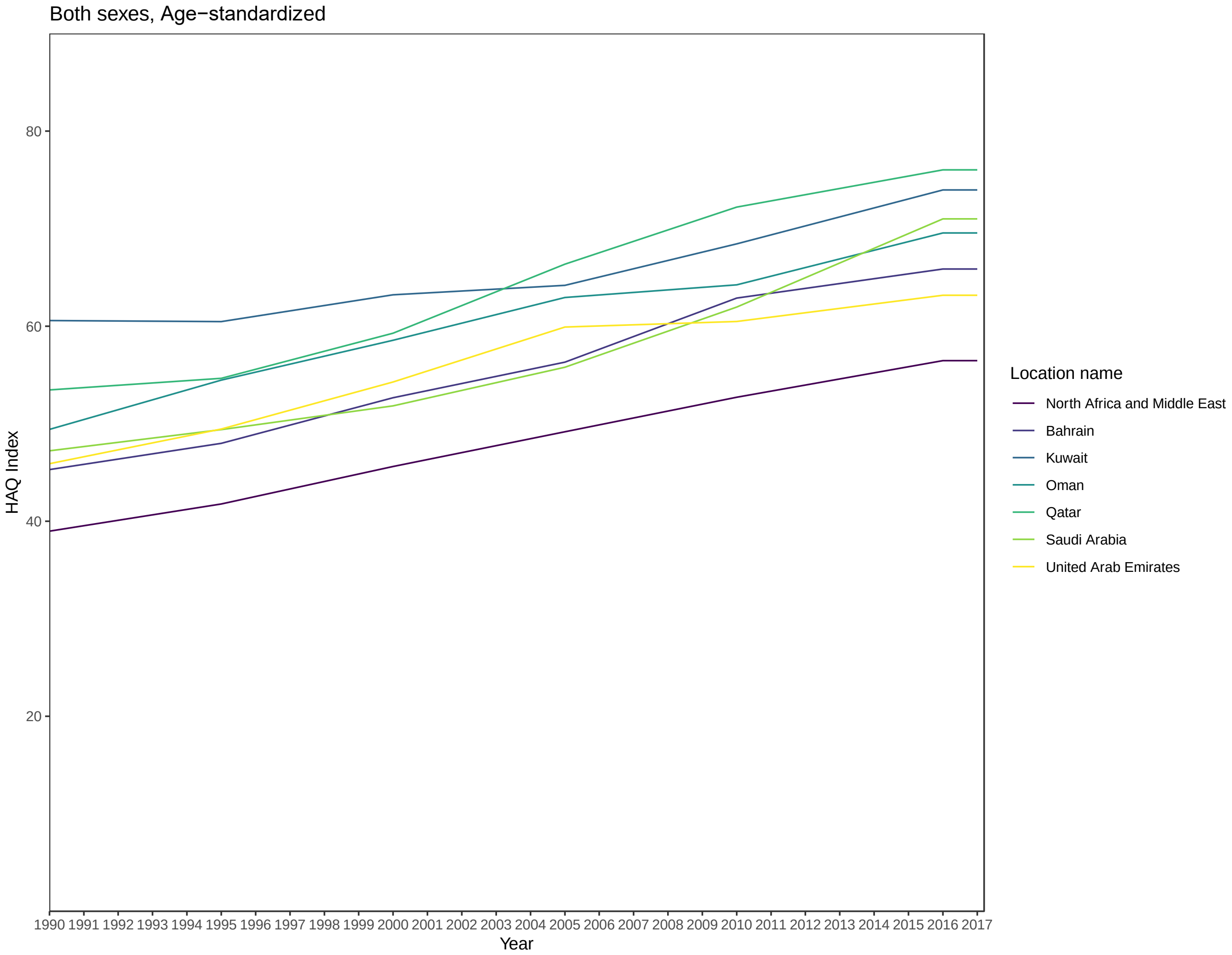

The Gulf Cooperative Council countries are Saudi Arabia, Bahrain, Kuwait, Oman, Qatar, and the United Arab Emirates.
